# Proton beam therapy causing pericarditis – a rare case of radiation induced cardiotoxicity

**DOI:** 10.1186/s40959-022-00135-0

**Published:** 2022-04-18

**Authors:** Rahul Gupta, Muling Lin, Gary M. Freedman, Deborah W. Sundlof, Cheri Silverstein Fadlon

**Affiliations:** 1 Lehigh Valley Heart Institute, Lehigh Valley Health Network, Allentown, PA USA; 2 University of South Florida, Morsani College of Medicine, Tampa, FL USA; 3 Department of Radiation Oncology, University of Pennsylvania, Philadelphia, PA USA; 4 Lehigh Valley Heart Institute, Lehigh Valley Health Network, PA Bethlehem, USA

**Keywords:** Proton beam, Radiation, Pericarditis, Chest pain

## Abstract

Acute pericarditis is caused by the inflammation of the pericardium which can result in an effusion around the heart. Proton beam therapy causing radiation-induced pericarditis is not a well-known cause of pericarditis. We present a case of a patient with Li-Fraumeni Syndrome who developed acute onset pericarditis, presumed to be secondary to proton beam therapy.

## Background

Acute pericarditis is a relatively common cause of chest pain, accounting for approximately 5% of presentations of chest pain [1]. Inflammation of the pericardium can result in an effusion that compresses the heart, as well as causing fibrosis of the pericardium that leads to a constrictive syndrome [2]. The most common cause of acute pericarditis is viral, but there are other systemic causes of pericarditis including malignancy, autoimmune disease, and uremia. External causes such as medications (e.g., hydralazine, isoniazid), bacterial infection, and radiation should also be considered [3].

Li-Fraumeni Syndrome (LFS) is a hereditary disorder involving mutations of the tumor suppressor gene, TP53, causing a wide spectrum of childhood and adult malignancies [4]. Diagnosis of LFS is based on the classic LFS criteria of “Proband diagnosed with sarcoma before age 45 years AND first degree relative with a cancer diagnosed before age 45 years AND a first-degree or second-degree relative with any cancer with onset before age 45 years OR a sarcoma at any age” [5]. Once it is discovered that a patient is a carrier of the germline TP53 mutation, it is imperative that the patient undergoes routine cancer surveillance. In 2016, Villani et al. discovered the feasibility of a multi-modal screening protocol called the “Toronto Protocol” which showed significantly increased survival for patients with LFS [6]. Based on this protocol, it is important to monitor carriers with routine physical examination, blood work, and imaging studies for signs of active malignancies.

We present a case of a patient with Li-Fraumeni Syndrome who developed acute onset pericarditis, presumed to be secondary to proton beam therapy.

## Case presentation

A 46-year-old female was treated in 2014 for left breast cancer diagnosed in June 2014. She was started on neoadjuvant docetaxel, carboplatin, trastuzumab, and pertuzumab for 6 cycles, followed by maintenance chemotherapy from July 2014 to October 2014. She had a bilateral mastectomy in December 2014 and had an additional 6 months of adjuvant trastuzumab that was completed in June 2015. The patient did not have any interruptions in her planned therapy due to cardiac concerns. In August 2014, she was diagnosed with Li-Fraumeni syndrome after her son had an osteosarcoma and subsequent rhabdomyosarcoma and tested positive for LFS. Her genetic testing revealed a pathogenic c.592delG mutation in TP53, consistent with LFS. In 2020 she presented with a recurrence in the left reconstructed breast and axillary nodes. She underwent a local excision and had a 1.7 cm mass with a positive posterior margin, axillary node dissection showing four of nine nodes positive, and dose-dense doxorubicin, cyclophosphamide, and paclitaxel. She was recommended postmastectomy radiation for the four positive nodes and positive deep margin. She was treated to a standard dose of 50 Gy in 25 fractions. Proton beam radiation was chosen to minimize scatter dose to other organs given her risk for second malignancies and radiation-related sarcoma due to the Li-Fraumeni.

**Fig. 1:**
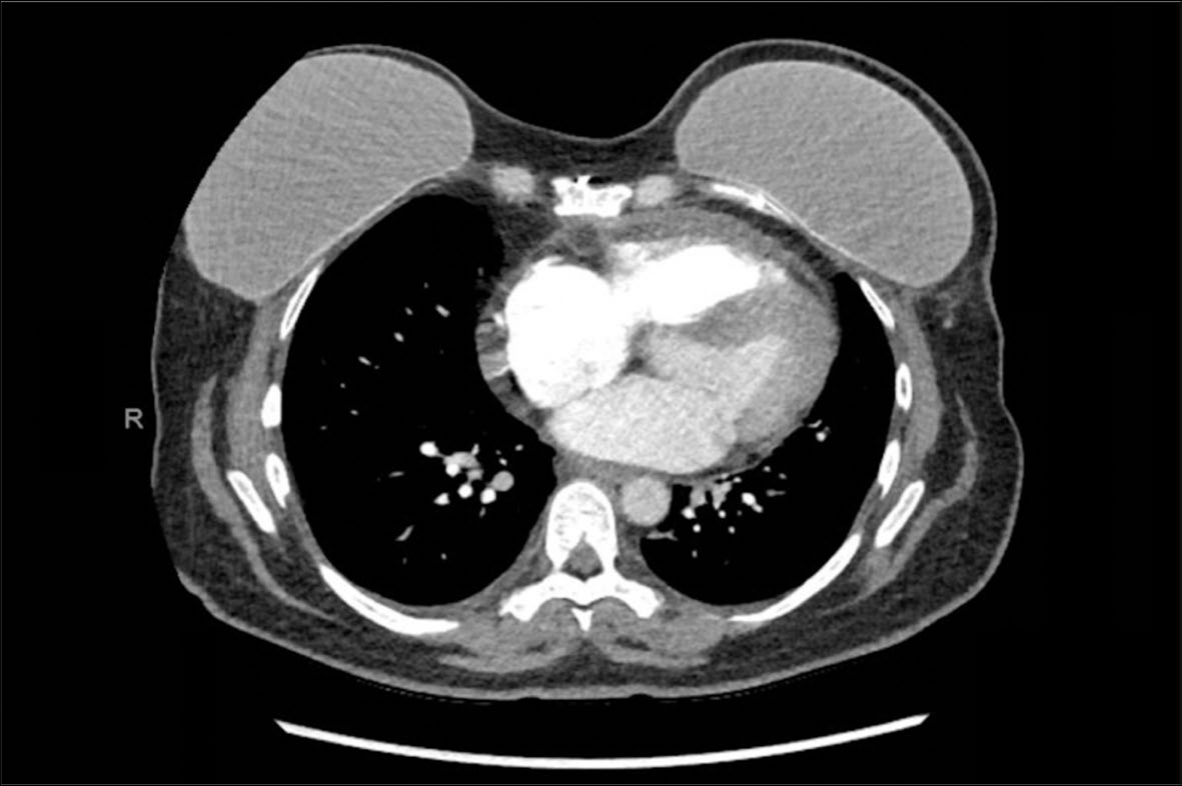
Chest CT showing mild pericardial effusion, no evidence of inflammation or cardiomyopathy

**Fig. 2:**
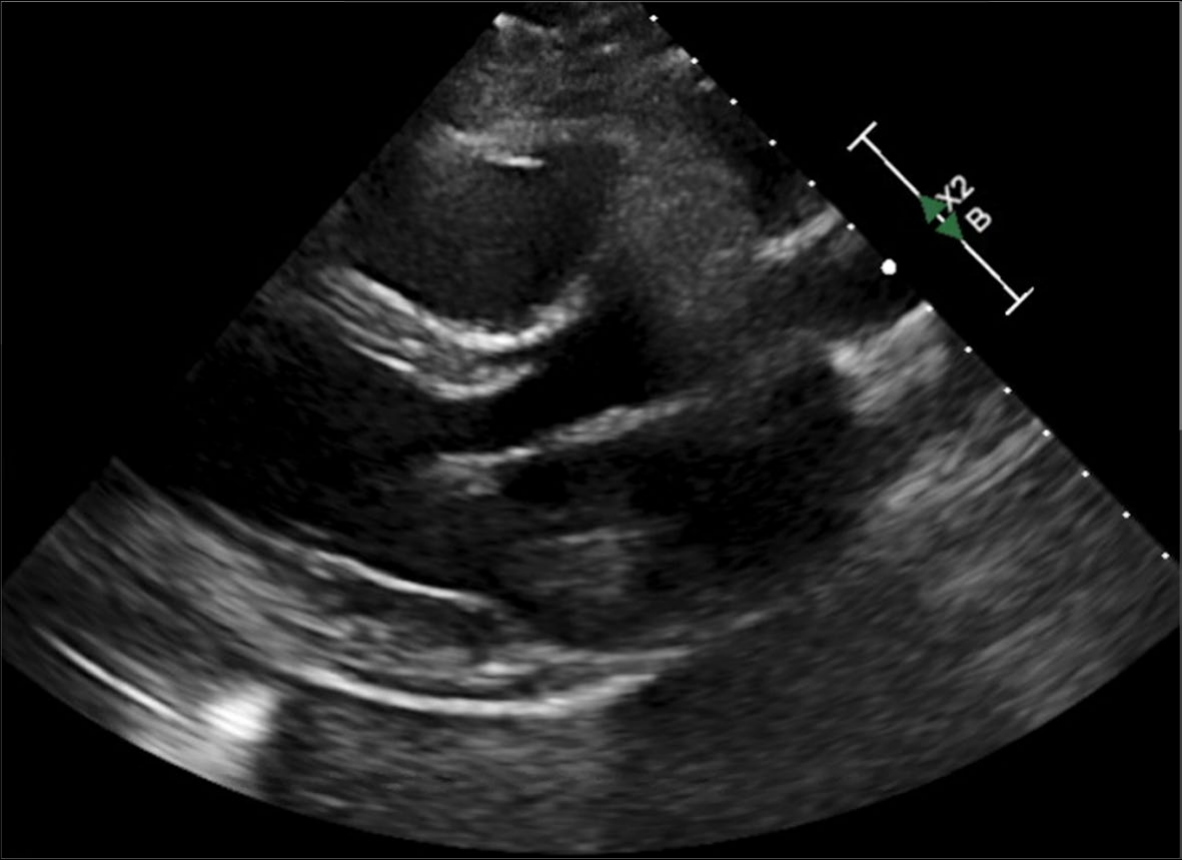
Echocardiogram showing trivial pericardial effusion

She presented with left-sided pleuritic sternal pain on 6/6/2021. She was recently started on proton radiation therapy on 5/25/2021 and had received 18 Gy of planned 50 Gy. Her chest pain was 5/10, worse with deep inspiration, and not alleviated with 200 mg ibuprofen and then 600 mg ibuprofen. She was referred to see her cardiology team 6/10/2021. Electrocardiogram (ECG) showed normal sinus rhythm with low voltage in limb leads with no ST changes or PR depression. Given her pleuritic pain and history of cancer, D-dimer was ordered and was slightly elevated at 0.77. CT chest was negative for any acute pulmonary embolism. Trace pericardial fluid was seen on the CT scan (Fig. 1) which was further evaluated with a repeat echocardiogram that showed a trivial pericardial effusion with no evidence of tamponade (Fig. 2). Her prior echocardiogram 6 months ago showed no evidence of pericardial effusion. Her ESR and CRP were significantly elevated at 38 and 145 respectively. The etiology of her chest pain was deemed to be secondary to pericarditis induced by proton beam therapy. While there was consideration that her chemotherapy regimen could have caused her pericarditis, the timeline and presentation of her symptoms were more likely due to the proton beam therapy as the precipitating cause. She was started on ibuprofen 800 mg three times a day with a scheduled taper. The patient continued to have persistent chest pain and was started on colchicine 0.6 mg daily with full resolution of her symptoms within a month.

## Discussion

The presence of LFS in this patient adds complexity to the case, as there have been recommendations against the use of radiotherapy in patients with LFS due to the risk of radiation-induced malignancies [7]. However, recent studies have shown that the incidence rate of radiation-induced malignancies in LFS patients may not be as high as previously believed. Therefore, the decision was made to proceed with proton beam therapy for this patient [8].

Proton Beam Therapy is a relatively new form of radiation used to treat cancers. Conventional radiation therapy utilized photon rays, which induced irreversible damage in the DNA of tumor cells, resulting in tumor cell death. However, there is associated normal tissue death with radiation. Newer targeted forms of photon radiation have been developed, but the risk of normal cell death and the occurrence of a second malignancy due to DNA damage are risks to be considered. Newer radiation techniques involve charged particle radiotherapy, which involves charged protons (H+) instead of photons [9]. Because charged protons have a very rapid energy loss in the last few millimeters of penetration, it allows for very precise localization of the radiation while minimizing the radiation-induced adverse effects on normal tissue [10].

**Fig. 3:**
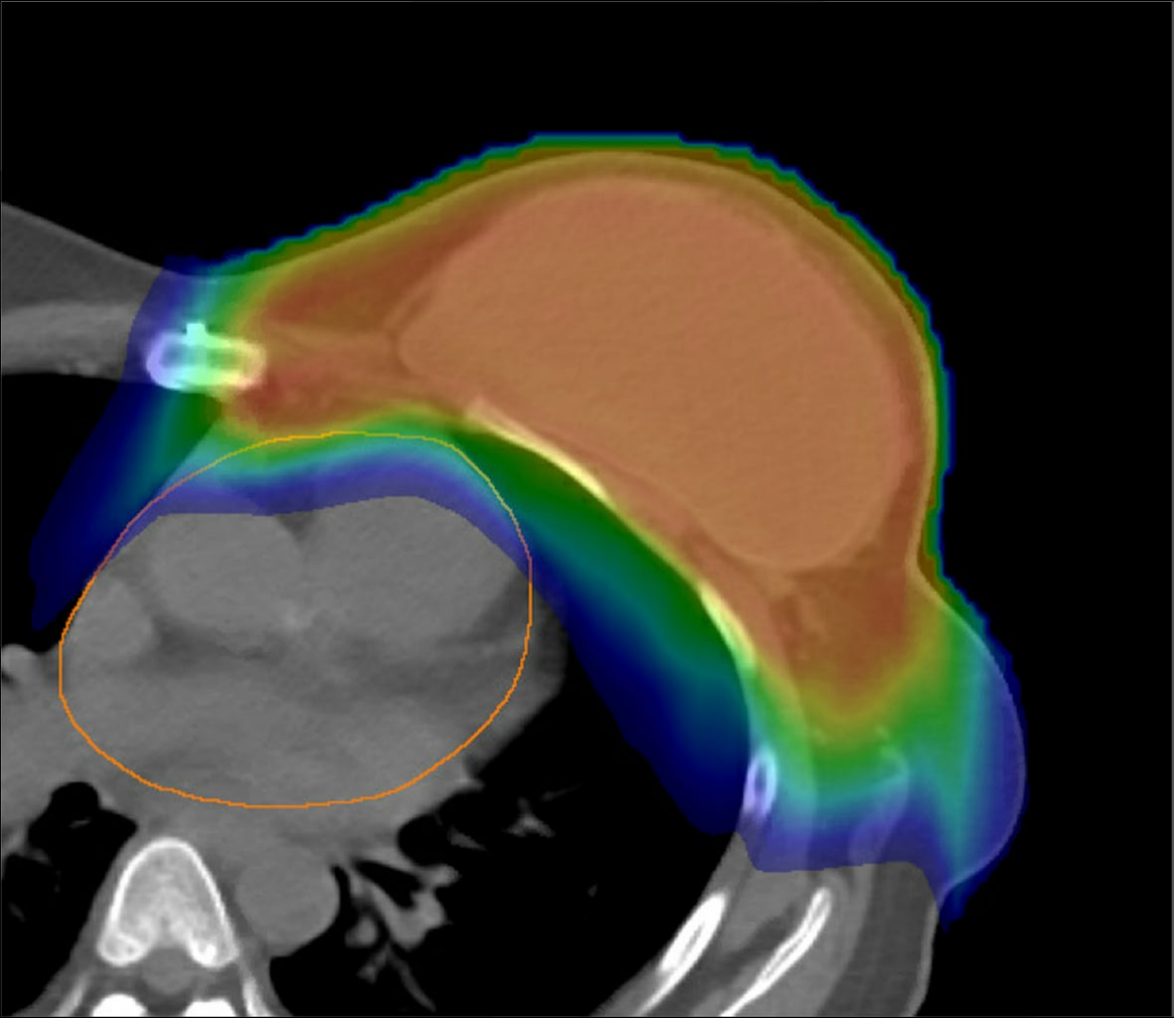
Colorwash of the proton isodose distribution on an axial slice at the level of the heart. The red represents the high prescribed dose intended for the target at risk for breast cancer, green mid-level dose, and blue low dose of the proton dose

Due to the improved localization of proton beam therapy, it is considered that all radiation-induced adverse effects would be decreased, though they can still occur. The decision to use proton therapy on this patient was predicated on mounting evidence of the benefit of proton therapy over standard photon therapy, as demonstrated by Stick et al., that proton therapy both reduced cardiotoxicity and reduced recurrence of breast cancer when used to treat primary breast cancer, when compared to photon therapy [11]. The proton plan resulted in a mean heart dose of 200 cGy, mean left lung dose of 792 cGy, and minimal dose to the opposite breast and lung – much lower than would be expected from comparable photon radiation plans due to the physical properties of the charged protons compared to x-rays. There have been several cases documented of radiation-induced pericarditis although rare in breast cancer [12–14]. However, on review of literature, proton beam therapy is not a well-known cause of pericarditis [15]. Cardiac side effects should theoretically be rarer with protons based on the calculated mean dose, and in this case in particular her symptoms began early on in the full course of treatment. However, there is a theoretical risk for an increase of side effects at the distal edge of the proton beam where increased linear energy transfer can occur and increased relative biological effect. For example, rib fractures at the distal edge of proton beams have been reported [16]. In this patient, the distal edge of the protons was by intention made to cover the chest wall at risk for breast cancer but stop at the edge of the pericardium to avoid the heart (Fig. 3). Further research in an ongoing phase III prospective trial comparing proton and photon beam radiation for breast cancer may give additional data on whether these kinds of distal range complications are more common than appreciated [17].

Diagnosis of radiation-induced pericarditis is similar to acute pericarditis, with some key differences to consider. As per European Society of Cardiology 2015 guidelines, a formal diagnosis of acute pericarditis requires two of the following: 1) Typical pericardial chest pain, 2) Pericardial rubs, 3) New widespread ST elevation and/or PR depression, 3) New or worsening pericardial effusion [18]. Our patient had pericardial chest pain along with new pericardial effusion, along with elevated inflammatory markers of ESR and CRP in the setting of active radiation treatment, which was strongly suggestive of radiation-induced pericarditis.. For any cancer patient with pericarditis, further consideration must be given in light of an underlying malignancy. CT scan should be ordered to rule out any emerging metastasis, and biopsy of the pericardium and/or cytology of the fluid could be performed if clinical suspicion is high for malignancy [19].

Radiation-induced pericarditis is treated with the first-line treatment with non-steroidal anti-inflammatory drugs (NSAIDs). No evidence demonstrates the clinical benefit of one NSAID to another, so ibuprofen was chosen for this patient due to its relatively low adverse effect profile. However, due to a lack of response to conventional treatment, colchicine was added to her NSAID regimen. This decision was based on a randomized controlled trial that evaluated Colchicine and NSAID vs. conventional NSAID therapy, which found that the addition of colchicine significantly reduced the risk of incessant/recurrent pericarditis and reduced the rate of symptom persistence [20]. Glucocorticoids can also be considered but are typically only recommended when the underlying cause is due to connective tissue disease, autoimmune conditions, or uremia [18]. They are not recommended for other causes of pericarditis due to the significant risk of adverse effects.

## Conclusion

This case represents a rare side effect of radiation-induced pericarditis which was most likely related to proton beam therapy. A high index of clinical suspicion and early management strategies can lead to rapid symptomatic improvement in the patients.

## Data Availability

Data sharing is not applicable to this article as no datasets were generated or analyzed during the current study.
